# The Validity of a Dual-Force Plate for Assessing Counter-Movement Jump Performance

**DOI:** 10.3390/s24175748

**Published:** 2024-09-04

**Authors:** Chuangui Mao, Ming Li, Xinglu Li, Zhengao Li, Tao Liu, Liangsen Wang, Wenfei Zhu, Lixia Chen, Yuliang Sun

**Affiliations:** School of Physical Education, Shaanxi Normal University, Xi’an 710119, China; maocg117@snnu.edu.cn (C.M.); 15921214889@163.com (M.L.); lixinglu@snnu.edu.cn (X.L.); lizhengao@snnu.edu.cn (Z.L.); liutao0604@snnu.edu.cn (T.L.); wlsen13@snnu.edu.cn (L.W.); wzhu@snnu.edu.cn (W.Z.)

**Keywords:** validity, force plate, CMJ

## Abstract

The objective of this study was to assess the concurrent validity of the Kunwei force plate system in relation to variables during a counter-movement jump (CMJ) task, in comparison to the Kistler in-ground force plate system, which is considered the “gold standard”. Methods: In a single testing session, the Kunwei force plates were placed directly on top of the in-ground Kistler force plate. This allowed for the simultaneous collection of vertical ground reaction forces from 30 participants (male, age = 22.8 ± 2.8 years, body mass = 74.3 ± 12.3 kg, height 178.2 ± 4.6 cm) during CMJ tests. The consistency between force plate systems was assessed using ordinary least products regression (OLPR) with bootstrapped 95% confidence intervals and the Interclass Correlation Coefficient (ICC). Results: There was no fixed or proportional bias in the CMJ variables measured between the force plate systems. The variables exhibited a strong correlation across the force plates during the CMJ task (ICC > 0.950, *p* < 0.01). Conclusion: The findings of this study indicate that there was no statistical difference between the Kunwei and Kistler force plate systems when evaluating common CMJ strategy and outcome variables, which are considered the gold standard. Hence, the Kunwei force plate can be regarded as a reliable substitute for the established industry benchmark in evaluating the force–time characteristics of the CMJ. Researchers, athletes, and coaches have the option to utilize this affordable and portable choice as a substitute for the more expensive laboratory-based force plate system. This alternative allows for the precise measurement of CMJ performance and force–time variables.

## 1. Introduction

The force plate is the most commonly used type of force transducer in sports and clinical biomechanics [1]. It has been widely used in healthcare and research applications [2,3,4]. Millions of force plate tests are performed each year because they provide fast, portable, and accurate information through the relevant professional tests for participants without additional complex technology, such as motion capture systems, electromyographs, etc. [5].

Force platforms use transducers to measure the forces applied to them in different dimensions [1]. The following transducer types are frequently seen in force plates: Hall Effect, piezoelectric, capacitance, strain, and piezoresistive sensors. While the technological aspects of these sensors differ, they always monitor force on an electrical current that flows through them and document any variations in that current when a force is applied. The platform’s calibration is then used to convert the change in current into a Newton value. There are still limits on the price and mobility of force transducers and force plates that are commercially available for use during sports. There are not many economical portable force plates suitable for various environments, allowing for easy installation and removal when travelling for competitions or training sessions [6]. Especially given the price, convenience, environment, and other factors, the in-ground force plate in the laboratory is limited despite its higher accuracy. Meanwhile, new portable dual-force plate systems are being introduced commercially. They can enhance the performance of individuals and make force measurements easier for researchers, coaches, and athletes to acquire. Past studies have been conducted to verify the in-ground and portable force plates in laboratories to prove the accuracy and reliability of the tested force plate [6,7,8,9].

However, the previous studies have shortcomings in terms of experimental design and statistical methods. Some researchers failed to control the details of experimental movements strictly. For example, they could not design or describe the content of warm-up clearly in previous research. A standard warm-up must consist of an aerobic, dynamic stretching, and skill rehearsal component [10]. Otherwise, it may not allow participants to achieve a counter-movement jump with maximal effort [11]. Moreover, only using high correlations to verify the study’s accuracy cannot indicate strong agreement between the two methods [12]. The statisticians suggest that disagreement or bias should be prioritized over agreement between methods or measures. Estimates of disagreement are crucial for two key reasons: (1) to compare a particular method or measurement to another and (2) to identify the fixed and proportional bias [13,14]. When one method consistently yields higher (or lower) data than those obtained from the other method, it is said to have a fixed bias [15]. Proportional bias refers to a situation where one method gives numbers that are consistently higher or lower than those obtained from other methods, with the difference directly proportional to the magnitude of the measured variable [15]. Therefore, data should be analyzed using appropriate statistical tests to support both comparisons [16]. In consistency test studies, the Bland–Altman approach is a frequently employed methodology. Instead, Ludbrook makes the case for comparing measuring techniques using ordinary least product regression (OLPR) [13,14,15,17]. According to the author, the OLPR is a sensitive technique that can detect the bias between fixed and proportional bias amongst approaches. It is also appropriate to calibrate a particular method against another.

This study aimed to determine the accuracy of the Kunwei force plate system by comparing its results to those obtained from a Kistler in-ground force plate system, which is considered the ‘gold standard’. This was carried out by assessing the consistency between specific variables during the CMJ trial. According to our hypothesis, OLPR analysis will reveal a high degree of consistency—free from fixed or proportionate bias—between the force plate systems with respect to all CMJ variables. In addition, we hypothesized that there would be a strong correlation between all variables measured by the force plates during the CMJ test.

## 2. Materials and Methods

### 2.1. Participants

Thirty adults (height 178.2 ± 4.6 cm, body mass 74.3 ± 12.3 kg, age 22.8 ± 2.8 years) with various sporting histories, including football, basketball, athletics, powerlifting, and more, were included. The participants were uninjured and capable of exerting maximum effort during the trials. They willingly chose to take part in the study and supplied written consent, which was accepted by the institutional ethics committee (No: 202316016 2023-09). We only pay attention to the agreement between the two force platforms. Hence, this study is not influenced by the participants’ present training status or their past experience with resistance and vertical jump training.

### 2.2. Design

**Figure 1:**
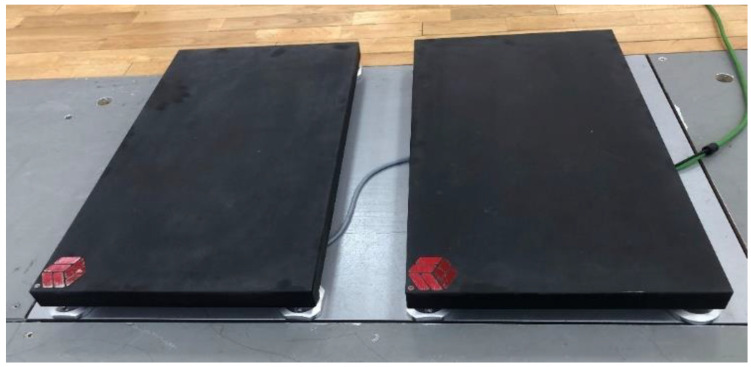
Example set-up for data collection.

This study uses a cross-sectional design, in which testing occurred in the sports biomechanics laboratory at the Shaan Xi Normal University in October 2023. Before the test, a standard warm-up (10 min) was conducted to reduce the risk of injury for each participant. It includes jogging, dynamic stretching, self-weight full squats, and submaximal CMJs. To measure the ground reaction force (GRF) generated by each leg separately and at the same time with a frequency of 1000 Hz, we used the Kunwei resistive force plate (KWYP-FP6035, Kunwei Sport Technology Co., Ltd., Shanghai, China), and its system includes two portable force plates. It is placed directly on top of and within the dimensions of a piezoelectric in-ground force plate (9287CA, Kistler, Winterthur, Switzerland), as shown in Figure 1.

The GRF data were collected for five seconds during counter-movement jumps (CMJs) using their own software (KW3.0.10.9M, Kunwei, Shanghai, China) and Mars software (V5.2, Kistler, Winterthur, Switzerland) for the two distinct force plate systems, respectively. Before each trial, both systems were zeroed to exclude the influence of the Kunwei system’s weight on the Kistler system (considered the gold standard) during data collection.

### 2.3. Counter-Movement Jump

The counter-movement jump is a type of vertical jump frequently utilized to assess the maximum anaerobic power production of the lower limbs. It can monitor an individual’s neuromuscular ability and reflect their physical readiness, which involves the synchronized extension of the trunk, hip, knee, and ankle [18].

The person is positioned near the center of the plate, with their feet either parallel or slightly turned outward. The distance between the feet should equal the breadth of the shoulders. The subject places their hands on the hips. Before starting a test, select input parameters. Then, the subject acquires an upright stance position. Each person positioned themselves on the force plates and remained still for at least one second before performing maximum effort trials (repeated three times) upon receiving a “3, 2, 1, jump” instruction. The participants were instructed to leap with maximum speed (referred to as strategy) and height (referred to as outcome) [17]. The purpose of standardizing performance with this cue was to motivate the participants to execute trials to achieve the highest possible height while emphasizing the importance of maintaining a fast movement during the unweighting, braking, and propulsive phases (i.e., a short time to take-off). This was to prevent them from independently favoring a strategy that prioritizes speed or height [18]. The software will autonomously detect the starting point and conclusion of the jump and instantly cease the collection of data. Participants performed three counter-movement jump (CMJ) trials with 60 s of rest between each session.

**Figure 2:**
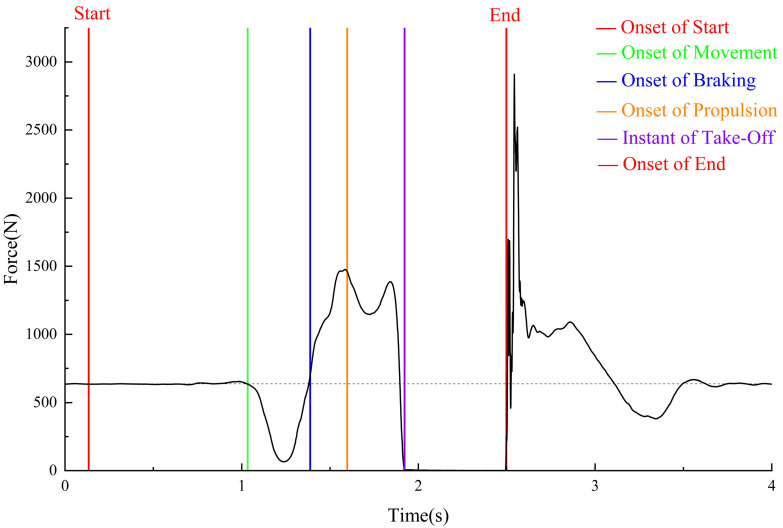
A representative example of a counter-movement jump trial and the occurrence of key events.

Prior to analyzing any data in the force plate system, coaches and sports scientists must have a comprehensive understanding of the CMJ force–time curve. It will facilitate comprehension of the fundamental stages and indicators from which variables are derived. Identifying the CMJ phases was recently accomplished utilizing the methodologies outlined in the existing literature [19,20]. The essential events are delineated in Figure 2, while the chosen indices of CMJ are displayed in Table 1.

Force–time data were not filtered and were processed in a customized spreadsheet [21,22]. The six key phases of CMJ (weighing, unweighting, braking, propulsion, flight, and landing) were defined and derived from force–time records in previous studies [19].

The initial phase of the CMJ is the weighing phase, also known as the silence period or stance phase. The athlete is required to maintain complete stillness during this phase [19]. The body weight was determined by calculating the average force exerted throughout a period of at least 1 s while in the position of ‘calm standing’. The laboratory force plate system was zeroed before each trial to eliminate the influence of the portable force plate weight [23,24,25,26].

The quiet standing force’s standard deviation (SD) was computed, and a threshold of body weight ±5 SD was established as the starting point. Data processing began 30 ms before this time, as previous studies indicate that the subject remains still at this phase and the assumption of zero velocity remains unaffected [21]. The identification of take-off involved three stages: firstly, the determination of the first force value that was less than 10 N and the subsequent force value that was greater than 10 N [27]; secondly, the identification of points 30 milliseconds (MS) before and after these points to define the center of the flight phase; thirdly, the mean and standard deviation of the force during the flying phase are calculated, and the mean force +5 SD are used to determine take-off [8].

The subsequent event is the flight phase, during which the athlete departs from the force platform in order to achieve the highest possible vertical displacement. This phase begins when the force during take-off drops below a specific threshold and concludes when the athlete makes contact with the platform again, and the force increases beyond a specific threshold. In a recent study, a threshold of force equivalent to 5-times the standard deviation of flight force (measured while the force platform is not carrying any weight) was effectively employed to detect take-off and touchdown. This threshold is applied to a 300 MS segment of the flight phase [23,24,28]. It is worth noting that when determining leap height based on flight time, it is assumed that the peak of the jump happens during the flight time period. However, this assumption only holds if the height of the center of mass (COM) is equal at the moment of take-off and landing [29,30]. Therefore, it is recommended that researchers utilize the take-off velocity approach (impulse-momentum theorem, as shown in Table 1) to estimate jump height [25].

### 2.4. Statistical Analysis

We used SPSS 25.0 (Statistical Package for Social Sciences software 25.0, IBM, New York, NY, USA) for data analysis during the force plate verification experiment. The test methods used are ordinary least product regression (OLPR) and Interclass Correlation Coefficient (ICC). It can reflect the degree of correlation and consistency between the measurement results of two measuring devices [8] and calculate their proportional and fixed deviations [7]. The consistency between force plate systems was assessed using the OLPR method instead of the Bland–Altman approach. This choice was made in accordance with the guidelines provided in the literature to date [15,17,31]. If the 95% confidence interval (CI) for the intercept, obtained through bootstrapping, does not include the value 0, it is concluded that there is a fixed bias. If the 95% CI for the slope, calculated using bootstrapping, does not include the value 1, it can be concluded that there is proportionate bias. The minimal detectable change (MDC) was calculated as a measure of the minimal amount of change that is not likely to be due to the variation in a measurement [32]. The repeated measures analysis of variance (ANOVA) with Bonferroni post hoc corrections for different variables was used to identify systematic bias for 3 CMJs.

## 3. Results

Table 2 shows the descriptive statistics, OLPR coefficients, and the related bootstrapped 95% confidence ranges. No consistent or proportional bias was observed between the two force plate systems for the CMJ variables examined. Table 3 shows a strong correlation between all variables measured by the force plates during the CMJ test (*p* < 0.01). The Shapiro–Wilk test shows that each group’s data were normally distributed (*p* > 0.05). According to Mauchly’s sphericity test, the dependent variable’s variance–covariance matrix is equal. All outcome measures except Absolute Maximal Force have no statistically significant differences. Bonferroni multiple mean comparison results show that the difference between the first and second trial of the Absolute Maximal Force was statistically significant, and the difference between the first and third trial values was statistically significant, with corrections F (2, 58) = 6.622, *p* < 0.01, partial η^2^ = 0.186. See also Table 4.

## 4. Discussion

The objective of this study was to establish the concurrent validity of a portable dual-force plate system by comparing the consistency between specific measurements acquired during the CMJ test using the test system and those acquired with the Kistler in-ground force plate (gold standard) system for accuracy.

According to the findings of this study, the force plate system developed by Kunwei may be seen as an accurate choice for gathering data on CMJ and force–time measurements. This conclusion is based on the analysis of OLPR and ICC, which revealed no fixed or proportional bias between the two force plate systems. The study involved 30 participants; the details can be seen in Table 2.

In a previous study, three-trial averages were only calculated for each athlete’s velocity at take-off and vertical jump height of the CMJ [9]. However, the jump height does not explain which method was used to calculate. Discrepancies in reported jump height have arisen due to variations in mathematical techniques and equipment employed [33]. Based on the findings of the previous study of force plates, the former results will be higher than the latter [34]. Thus, the practitioners are encouraged to use the vertical velocity at take-off to calculate jump height. In addition, the investigations also evaluated and compared impulse, peak force, and time variables with the Kistler force plate, which is considered the gold standard [6,8]. Silveira et al. only included 2 participants, whereas Lake et al. used 28 participants. A similar study used an experimental design without control of the arms. The author emphasized that allowing subjects to swing arms during CMJ trials may have been a negative factor for jump height [9]. Consequently, the jump strategy significantly impacted the mechanical characteristics, with deep jumps resulting in higher jump heights and concentric velocities than shallow jumps [35]. Thus, the uniformity of test movement standards is essential.

From a drop jump (DJ) perspective, previous studies indicate disparities between the optimal height of a box and the actual distance it falls [36]. It would alter the velocity at which the touchdown happens and the specific features of the force–time during the braking phase. Therefore, utilizing the DJ could potentially impact the precision of prescribing this training load [7,37]. In addition, given the DJ’s strategy, a counter-movement DJ will produce a higher jump height than a bounce DJ. The experimental requirement is to perform the drop jump with little contact time while seeking to maximize leap height, which may present a paradoxical situation, as in a previous study [7]. Therefore, in our experiment, we selected the CMJ and conducted a comprehensive analysis by incorporating outcome variables from the test. The results also justified these analyses, which recognized no fixed and proportional bias for the comparison in our study. These results support a previous study’s conclusions with a similar approach [7]. Moreover, our study utilized a more stringent methodological and statistical design approach to demonstrate a stronger consistency between the two force plate systems.

For Silveira et al., the precision and accuracy of comparison were evaluated by assessing the peak force, time for acquiring peak force, and impulse [6]. Acquiring the corresponding data derived from the measurement of the gold standard is the first step towards validating the new method. As predicted, all factors exhibited a strong positive correlation. The statistical approach measures the extent to which two variables are connected, but a strong correlation does not necessarily indicate a strong agreement between two methods. This is because it only analyzes the linear relationship between two sets of observations [12,38]. In other words, the use of correlation is misleading [12].

If measurements have been taken continuously, the two primary options are the Bland–Altman method of differences and ordinary least product regression (OLPR) analysis. Several declare that the former method is easy to carry out but fails to differentiate between fixed and proportional bias accurately. The OLPR analysis, despite its higher level of complexity, successfully accomplishes this objective. In addition, the consensus among biostatisticians is that the Pearson product–moment correlation coefficient (r) is often considered to be ineffective in detecting bias [14]. Therefore, we opted for our study’s more reliable OLPR analysis based on recommendations from previous research. OLPR is a simple and powerful tool for detecting systematic disagreement and perhaps the only “philosophically” correct statistical technique [7,31]. This choice was made instead of using Pearson’s correlation coefficients and Bland–Altman plots with 95% limits of agreement (LOA), which have been used in other studies [6,8,9,39].

Despite the widespread usage of force plates in laboratories, their applicability during sporting activity is still limited by factors such as accessibility and cost. Several affordable portable force plates with certain weights and dimensions are designed to enhance their usability in various settings, enabling easy set-up and removal while travelling for contests or training sessions [6]. The primary factors influencing the selection of this specific portable force platform are its portability, lightweight design, and affordability, which are advantageous for sports biomechanics. The utilization of portable force plate systems in sports science and technology can greatly improve the scientific rigor and effectiveness of sports training. According to this study’s findings, the Kunwei system is a reliable and inexpensive alternative to conventional in-ground force plate systems for conducting the CMJ test. It offers accuracy and portability while being more affordable. The outcomes of this study might provide practitioners with valuable insights into the advantages and constraints of portable force plate technology.

## 5. Conclusions

This study’s results indicate no fixed or proportional bias between the Kunwei and Kistler force plate systems when assessing common factors related to the CMJ. Therefore, the Kunwei system can be seen as a reliable substitute for the widely accepted industry standard. Researchers, athletes, and coaches can utilize this affordable and portable force plate system as a substitute for the more expensive laboratory-based force plate system. This alternative allows for the precise performance measurement and force–time variables of CMJ.

## Figures and Tables

**Table 1:** Selected force–time variable calculations for CMJ.

Variables	Definition	Calculation
Body weight (kg)	Body weight in a steady state	m = F/g
Jump Height (m)	The height of the jump is calculated from the Take-Off velocity.	$H=v^{2}/2g$
Absolute Maximal Force (N)	Maximal Force during the propulsion phase.	F = F_max_
Absolute maximal Power (P)	Maximal Power during the propulsion phase.	P = P_max_
Vertical Take Off Velocity (m/s)	The vertical movement velocity was calculated from Flight Time at the Take-Off.	v= $\frac{\int \left(F\left(t\right)-G\right)dt}{m}$
Jump Time (s)	The time interval between the movement and the take-off phase.	t = t_take-off −_ t_onset of movement_
Push Off time (s)	The time interval between the propulsion and the take-off phase.	t = t_take-off −_ t_onset of propulsion_
Flight Time (s)	The time interval between the take-off and the end phase.	t = t_end −_ t_take-off_
Push Off Force Impulse (N·s)	Force impulse between the propulsion and the take-off phases.	I = F · (t_take-off −_ t_onset of propulsion_)
RSImod	Jump Height divided by Jump Time.	RSImod = H/(t_take-off −_ t_onset of movement_)

Key: G, body weight; g, 9.8 m/s^2^; P, power; m, meters; s, seconds; N, Newtons; RSImod, reactive strength index modified.

**Table 2:** Descriptive and agreement statistics for the CMJ variables between Kistler and Kunwei force plates.

**Variables**	**Kistler** **(Mean ± SD)**	**Kunwei** **(Mean ± SD)**	**Intercept** ( 95% CI )			**Slope** ( 95% CI )		
Body weight (kg)	74.27 ± 12.27	74.01 ± 12.30	(0.35	**0.5**	0.65)	(1.00	**1.00**	1.00)
Jump Height (m)	0.39 ± 0.08	0.39 ± 0.08	(0.01	**0.01**	0.01)	(0.98	**0.99**	1.01)
Absolute Maximal Force (N)	1820.21 ± 337.30	1812.75 ± 338.71	(9.84	**14.46**	19.08)	(0.99	**1.00**	1.00)
Absolute maximal Power (P)	3954.3 ± 697.46	3960.86 ± 692.30	(−54.73	**−13.28**	28.17)	(0.99	**1.00**	1.01)
Vertical Take Off Velocity (m/s)	2.78 ± 0.30	2.73 ± 0.28	(0.07	**0.1**	0.12)	(0.98	**0.98**	0.99)
Jump Time (s)	0.90 ± 0.129	0.86 ± 0.12	(0.07	**0.08**	0.08)	(0.95	**0.96**	0.96)
Push Off time (s)	0.29 ± 0.03	0.29 ± 0.03	(0.01	**0.01**	0.01)	(1.00	**1.00**	1.00)
Flight Time (s)	0.57 ± 0.06	0.57 ± 0.06	(0.01	**0.01**	0.01)	(1.00	**1.00**	1.00)
Push Off Force Impulse (N·s)	202.17 ± 34.24	202.1 ± 34.53	(−1.67	**0.05**	1.76)	(0.99	**1.00**	1.01)
RSImod	0.44 ± 0.12	0.46 ± 0.13	(−0.05	**−0.04**	−0.03)	(1.02	**1.04**	1.06)

Key: RSImod, reactive strength index modified.

**Table 3:** Intraclass correlation efficient of CMJ variables between Kistler and Kunwei force plates.

Variables	ICC	*p*	MDC
Body weight (kg)	0.999	0.001	0.71
Jump Height (m)	0.958	0.001	0.03
Absolute Maximal Force (N)	0.999	0.001	21.59
Absolute maximal Power (P)	0.980	0.001	196.81
Vertical Take Off Velocity (m/s)	0.955	0.001	0.12
Jump Time (s)	0.972	0.001	0.04
Push Off time (s)	0.992	0.001	0.01
Flight Time (s)	1.000	0.001	0.00
Push Off Force Impulse (N·s)	0.986	0.001	8.11
RSImod	0.960	0.001	0.05

Key: RSImod, reactive strength index modified. MDC, minimal detectable change.

**Table 4:** The comparison of three CMJs variables for Kunwei force plates.

Variables		Test		F		*p*	
	1st	2nd	3rd		1st & 2nd	1st & 3rd	2nd & 3rd
Body weight (kg)	74.02 ± 12.52	73.97 ± 12.45	74.051 ± 12.57	0.325	1.000	1.000	1.000
Jump Height (m)	0.38 ± 0.09	0.38 ± 0.08	0.39 ± 0.08	2.186	1.000	0.268	0.115
Absolute Maximal Force (N)	1774.35 ± 318.63	1823.34 ± 343.90	1840.57 ± 365.77	6.622	0.010 *	0.018 *	1.000
Absolute maximal Power (P)	3940.87 ± 673.71	3958.23 ± 727.17	3983.47 ± 709.79	0.601	1.000	0.978	1.000
Vertical Take Off Velocity (m/s)	2.71 ± 0.31	2.73 ± 0.28	2.76 ± 0.29	2.070	1.000	0.313	0.108
Jump Time (s)	0.89 ± 0.14	0.85 ± 0.12	0.85 ± 0.11	2.004	0.390	0.416	1.000
Push Off time (s)	0.29 ± 0.03	0.29 ± 0.03	0.29 ± 0.03	1.850	0.226	1.000	0.208
Flight Time (s)	0.56 ± 0.07	0.57 ± 0.06	0.57 ± 0.06	4.681	0.268	0.057	0.186
Push Off Force Impulse (N·s)	200.30 ± 34.21	201.93 ± 35.6	204.07 ± 35.38	2.937	1.000	0.137	0.113
RSImod	0.44 ± 0.12	0.46 ± 0.14	0.47 ± 0.13	3.135	0.294	0.132	1.000

Key: RSImod, reactive strength index modified. *, *p* < 0.05.

## Data Availability

The data presented in this study are available on request from the corresponding author. The data are not publicly available for reasons of confidentiality.
